# The physiology of interoception and its adaptive role in consciousness

**DOI:** 10.1098/rstb.2024.0305

**Published:** 2025-11-13

**Authors:** Jacques Singer, Antonio Damasio

**Affiliations:** ^1^ Brain and Creativity Institute, University of Southern California, Los Angeles, CA 90089, USA

**Keywords:** consciousness, interoception, homeostasis, feelings, myelin, volume transmission, ephaptic coupling, vagus nerve, nucleus tractus solitarius, blood–brain barrier

## Abstract

The interoceptive nervous system uses signalling mechanisms distinct from those of exteroceptive, voluntary motor, cognitive and linguistic processes. While interoception relies mainly on analogue-like processing, the other systems favour digital-like signalling. This physiological feature allows interoception to map the state of the organism’s internal milieu in real time, in stark contrast to the rapid, precise and time-sensitive processing of exteroceptive, proprioceptive, cognitive and linguistic signals. This distinction arises from the unique features of the interoceptive process, namely the unmyelinated and lightly myelinated nature of afferent interoceptive neurons, which facilitates non-synaptic signalling throughout interoceptive pathways, notably in those of the vagus nerve and of neural structures devoid of a blood–brain barrier. We propose that by continuously representing the body’s state, spontaneously conscious homoeostatic feelings constitute the foundational substrate of subjectivity and the grounding for consciousness. This idea builds on our prior work, which grounds consciousness in core biology rather than high-level cognition. Consciousness enables adaptive and protective responses that maintain homeostasis and secure life.

This article is part of the theme issue ‘Evolutionary functions of consciousness’.

## 1. Introduction

Feelings are conscious experiences that arise from the brain’s representation of the body’s internal state [1–5]. An important and fundamental class of feelings is of the homoeostatic variety. They are spontaneously conscious and emerge when there are significant deviations away from physiological balance, whether negative (e.g. hunger, thirst, hypoxia, fever) or positive (e.g. satiety, wellbeing). Other feelings, such as feelings of emotions (e.g. fear or excitement), represent patterned combinations of homoeostatic shifts in response to challenges to internal stability or opportunities to improve it (e.g. increased heart rate and metabolic demand when encountering a predator contribute to the feeling of fear).

This perspective on the phenomena of feeling emphasizes the primacy of homoeostatic feelings in the architecture of the mind. What we commonly refer to as the ‘conscious mind’ can be described as a collection of continuously flowing physiological events with distinct functional characteristics: (i) spontaneously conscious *homoeostatic feelings* that describe the current internal state of the organism (and should not be conflated with emotions); (ii) *perceptions* of the external world and of our own movements as a result of exteroceptive and proprioceptive processes; (iii) *reflections* on those perceptions and associated memories, including reasoning; and (iv) *linguistic translations* of any of the above components. The homoeostatic component of the conscious mind constitutes a *feeling mind*. The other components—exteroceptive/proprioceptive, the reflective and the linguistic—constitute what we call the *modern mind*.

Importantly, the homoeostatic feeling process is fundamentally distinct from the other dimensions of the conscious mind. It serves as a continuous, spontaneously conscious interface between the body proper and the nervous system, anchoring subjectivity and providing the core foundation for consciousness.

The fact that each homoeostatic feeling event is *spontaneously known* to the organism (i.e. conscious), provides the organism with the information necessary to regulate and maintain life. If feelings were not spontaneously known, they would be unable to guide adaptive responses and their functional purpose in guiding behaviour would be different, or even non-existent.

The fundamental feature here concerns the kind of neurons responsible for such feelings. The goal of this article is to describe the distinct mechanisms through which homoeostatic feelings are generated. This leads to an account of the particular physiology responsible for interoception—the process that enables vigilant homeostatic regulation and thus supports survival. We also examine how interoception provides the foundations for consciousness, understood here as an adaptive, continuous feeling of the living organism.

Typically, the axons of neurons that support homoeostasis and affect lack myelin. This feature opens them to a variety of physiological events that the myelinated neurons supporting the exteroceptive, reflective and linguistic processes cannot engage in. In the absence of myelin, the neurons responsible for the world of feeling are anatomically and functionally able to *directly interface* with the surrounding structures of the body proper. Rather than using classical and physiologically elaborate synapses—which isolate the nervous system from the surrounding body structures—non-myelinated neurons rely on simpler physiological arrangements that enable ample communication between the nervous system and non-neural body structures. In this article, we describe such structures and operations and provide examples of the main physiological arrangements behind the generation of homoeostatic feelings. Those arrangements include volume transmission (direct chemical signalling), ephaptic coupling, the signalling conditions created by the lack of blood–brain barrier, and the signalling in the vagus nerve system—the tenth cranial nerve, which relays interoceptive information from throughout the body to the brain (see §4b). In each of these arrangements, the body proper and the nervous system *merge*, one literally sliding onto the other. As a result, whereas the neurons that support the exteroceptive/reflective and linguistic processes help produce a *sketch* and *constitute imagetic patterns*, such as those generated by vision or hearing, the neurons supporting *homoeostatic feelings* interface directly with body structures and generate an actual mixture of signals from the body proper and the neurons themselves. A term such as ‘*comingle*’, which we are introducing, seems appropriate to describe the making and the nature of this peculiar mixture. In brief, from a physiological standpoint, we do not have a singular kind of mind, but rather two kinds: a *feeling mind,* which continuously represents the state of life inside our organism as it copes with its intrinsic functions and with the world surrounding it; and the remainder of the representational processes, which are grounded on exteroception, proprioception, reflections on both, and the linguistic translation of the related imagery. As noted, we have proposed to call this latter process the *modern mind* because of its later evolutionary vintage.

The distinct physiology of interoception supports ‘analogue’ signalling alongside the predominantly ‘digital’ signalling of myelinated systems involved in higher-order exteroceptive and reasoning operations. Analogue systems convey information continuously—as real-valued signals—while digital systems do so binarily, using discrete states. Early computing relied on analogue designs for their flexibility, but digital systems eventually became dominant owing to their speed. Recently, however, the energy cost of complex digital systems—especially compared with the extreme efficiency of the brain—has revived interest in analogue approaches [6–8].

## 2. Digital-like, rapid and reliable signals support the modern mind

The exteroceptive, reflective and language processes rely on the presence of myelinated neurons and synapses for rapid, optimal and ‘noise-resistant’ neurotransmission. In addition to its insulating properties—which increase signal speed and efficiency—myelin serves as a physical barrier, shielding axons from their extracellular environment and thus preventing unintended modulation from neighbouring neural and non-neural tissues [9,10]. By enabling fast, all-or-nothing signalling, myelin allows action potentials to function as binary inputs for digital-like computations, such as those describable by Boolean logic [11,12]. Myelinated circuits process information in discrete, time-sensitive units that maximize precision and reduce noise. Digital processing is rapid and transforms information via integration within circuits, enabling complex logical computations [13,14]. Theoretical neuroscience has leveraged the parallels between digital systems and discrete neural signalling to model information processing in reflective, exteroceptive and linguistic functions. As in digital architectures, the brain organizes information flow hierarchically through circuit-like structures that perform progressive, time-sensitive transformations [15]. Myelination supports the speed, precision and synchrony required for such high-fidelity processing—enabling complex functions such as language, motor coordination and abstract reasoning [16–18]. These digital-like neural systems resemble deep learning models, where discretized signals pass through layered networks gated by activation functions.

Unlike the processes in the *feeling mind*, the exteroceptive, linguistic and reflective minds rely on rapid, discrete and highly precise signalling. Myelination and synaptic transmission are thus critical for the computational processes that support higher-order reasoning, language and exteroception—but not for interoception.

## 3. Analogue-like, continuous signals support the feeling mind

The feeling processes stand out owing to the extensive interaction with the body proper exemplified in autonomic visceral sensation from afferents in the vagus nerve [19,20]. Most afferent vagal interoceptive neurons are lightly myelinated (Aδ-fibres) or unmyelinated (C-fibres) [21] and are tightly ensheathed together by Schwann cells, in Remak bundles, which dispense with individual myelin sheaths [22]. Unlike myelin, these structures provide support and promote regeneration while preserving some axonal access to the extracellular environment and enabling direct biochemical communication with surrounding non-neural tissue and neighbouring unmyelinated cells [9,23,24]. *The absence of myelin and the close proximity of cells in the interoceptive system give rise to a seamless, highly interconnected and dynamic interface between the interoceptive system and the body proper* [25,26]. Unmyelinated fibres allow neurons to partake in various forms of non-synaptic signalling (NSS), including chemical and electromagnetic crosstalk, thus not depending on specialized synapses for interneuronal signal transmission.

As a result of lacking axonal myelin insulation, signalling in the interoceptive system is slow, continuous and sensitive to a broad range of graded inputs from the extracellular environment, such as ambient chemicals released locally by neighbouring neural or non-neural tissue, as well as from signals transmitted distantly through the bloodstream or cerebrospinal fluid [27,28]. This is in marked contrast to the ‘all-or-nothing’ logic of myelinated pathways, which prioritizes speed, precision and reliability [9,29]. The interoceptive system enables a continuous, diffuse and integrative sensing of internal bodily states [25,30]—a true communing of the nervous system with its own body. The slow and continuous integration of information over time in the interoceptive system depends on analogue signalling. In analogue systems, information is not strictly discretized into binary patterns such as spiking. Instead, it is represented through continuous, dynamic and graded signals. It enables steady sensing, providing the system with robust, adaptive information monitoring [31].

Analogue computation, in both computer science and biology, favours adaptability and continuity over precision and speed [32]. This continuously adapting information describes the core of interoceptive operations which constantly monitor graded information to generate continuous, real-time, dynamic representations of the body. But how exactly does this direct neural–non-neural interface give rise to analogue processing? The answer is contained in a collection of processes, which we discuss in the next section.

The heavy myelination of pathways in the *modern mind* confines information modulation and integration to synapses, thus favouring digital processing, although a small portion of neurons remain unmyelinated, allowing occasional instances of analogue signalling. It is likely that digital neural signalling evolved from analogue systems, sacrificing continuity and computational richness for greater conduction velocity and reliability in processing information from a rich, rapidly changing and uncertain environment.

## 4. The mechanisms of non-synaptic signalling

### (a) The neurobiology of non-synaptic signalling

#### (i) Volume transmission

Myelin acts as an insulating barrier that enhances the efficiency of signal transduction and enables saltatory signal conduction, where electrical signals jump from one node of Ranvier to another [33]. By confining ion exchange to these nodes, myelin maintains axonal integrity while reducing axonal permeability to molecules and agents present in the extracellular environment [25,34,35]. Once an action potential is triggered at the axon hillock, myelin facilitates its rapid and energy-efficient propagation through the circuit in which the neuron is embedded [36]. While external factors can still influence neuronal activity at the nodes of Ranvier, myelin drastically reduces signal modulation along the axon, ensuring high-fidelity transmission.

By contrast, unmyelinated and lightly myelinated axons lack this barrier and are exposed to agents present in their extracellular environment. This makes them more susceptible to ambient extracellular chemicals and allows them to engage in alternative forms of neural communication beyond typical synaptic transmission—non-synaptic signalling (NSS). For example, these axons can partake in volume transmission. Rather than being limited to rapid, point-to-point signalling, these neurons dynamically interact with their environment, integrating physiological cues from nearby neural and non-neural tissues. This allows more continuous, graded signal transmission [24].

The structural and functional differences between myelinated and unmyelinated neurons reveal fundamentally different information-processing methods in the brain. Myelinated axons, virtually sealed off from their environment by their insulation, rely on high-speed, highly localized synaptic transmission, whereas unmyelinated or lightly myelinated axons use additional alternative signalling pathways that favour continuous and broad integration over efficiency and rapidity.

A variety of neural specializations and structures facilitate these alternative NSS pathways in unmyelinated circuits. For instance, in the vagus nerve axons are tightly packed together in Remak bundles [22]. This facilitates inter-axonal interactions and promotes volume transmission which is critically dependent on distance [24]. Additionally, axonal varicosities—swellings along the length of unmyelinated axons—act as hubs for sensing synaptic release along the axons, participating in volume transmission and signal modulation along the axon. The presence of high-affinity receptors on these neurons further highlights their ability to continuously detect and respond to molecules at low concentrations in the extracellular space, solidifying their role in dynamic analogue sensing rather than all-or-none digital signalling [37]. Volume transmission may, for instance, enable unmyelinated axons to generate or reinforce subthreshold depolarizations that are too weak to trigger firing yet may travel along the axon, modulate neurotransmitter release in a graded, analogue manner and influence subsequent spikes. Consequently, some neurons may transmit both analogue and digital signals, challenging the view of axons as purely digital conduits [31,38,39]. Thus, subthreshold depolarizations provide an efficient means of transmitting information in a continuous and analogue manner, without solely requiring transformation into discrete spike-based codes such as rate coding, which is prominent in myelinated neurons. There may be other unidentified alternative mechanisms by which unmyelinated neurons transmit information.

An important consideration in the study of NSS is the role of the pharmacokinetics of non-synaptic compounds. Determining the half-life and diffusion range of these molecules in the extracellular space is key to understanding their non-synaptic activity. Some can only travel minimal distances, whereas others may travel longer distances over extended periods—some may even be transported throughout the organism via blood and cerebrospinal fluid [40,41]. Another key consideration is the kinetics and sensitivity of high-affinity non-synaptic receptors along unmyelinated axons.

*This neural–non-neural direct interface is at the core of interoception*. It enables neurons to integrate a wide range of different chemical information continuously, without depending solely on dedicated synaptic structures.

Despite NSS’s significant implications in signalling, few studies have characterized the molecules involved. Thus far, electrophysiological experiments have confirmed that adenosine triphosphate (ATP) [42], γ-aminobutyric acid (GABA) [43], serotonin [44–47], acetylcholine [48], vasopressin [49] and capsaicin [50] are involved in vagus nerve operations. A deeper neurobiological understanding of these compounds, their receptors and the analogue signalling they support in the interoceptive system could redefine our view of neural communication and reveal the full complexity of interoception. Recognizing NSS as a fundamental mode of direct brain–body communication is essential for advancing our understanding of interoception itself and of subjective awareness/consciousness (see §5) [3,51].

#### (ii) Ephaptic coupling

In tightly packed bundles of unmyelinated or lightly myelinated neurons—such as those in the vagus nerve—electromagnetic fields generated by one or more neurons can influence nearby firing activity through *ephaptic coupling* [52]. This process occurs at multiple levels—from shaping ion distribution to modulating membrane potentials and driving spatial–temporal synchrony in neural circuits [53]. Such NSS supports complex, graded, analogue-like processing across various timescales distinct from the digital-like computing found in the traditional, myelinated circuits involved in exteroception, cognition and language [52,54,55]. Ephaptic coupling has been demonstrated *in vitro* and *in vivo* as well as extensively studied *in silico*, in densely packed or in unmyelinated regions of the brain [53,56,57].

The interoceptive system possesses all the attributes required for ephaptic interactions. It is, thus, plausible that ephaptic interactions could enable continuous, graded, NSS, supporting complex analogue neural activation patterns essential for real-time internal state sensing and the construction of self-maps in the brainstem. Further research is needed to confirm the presence of ephaptic signalling in the interoceptive system and clarify its role in affective processes.

#### (iii) Blood–brain barrier fenestrations

In addition to the direct cellular neural–non-neural tissue communication between neural and non-neural tissue, the interoceptive system is composed of specialized central regions directly exposed to the brain’s vasculature. Typically, the brain’s vasculature is sealed by endothelial cells that tightly regulate the entry of bloodborne agents, shielding the majority of the brain from pathogens and preserving its intricate chemical balance. However, certain regions, known as circumventricular organs (CVOs), lack this barrier and allow free exchange between blood and neural tissue [58]. Although these fenestrations render these regions—and the brain—vulnerable, they enable rapid sensing of critical molecules (e.g. hormones, toxins and neuromodulators).

Many CVOs—including the area postrema, subfornical organ and median eminence—are closely connected to central interoceptive regions such as the nucleus tractus solitarius, the arcuate nucleus and the parabrachial nucleus [26,59]. The area postrema detects bloodborne toxins and may trigger emesis to prevent further toxin absorption; the subfornical organ senses blood salt levels and signals the hypothalamus to regulate kidney function to restore fluid balance [60]; the median eminence serves as the neural–non-neural interface for neuroendocrine signalling [61].

CVOs, thus, serve as sites of continuous central monitoring of internal states, enabling efficient homoeostatic regulation through direct interplay—comingling—between neural and non-neural systems [62].

### (b) The unique features of the vagus nerve system

The interoceptive system is composed of afferent pathways, central processing centres and efferent control systems. Key afferent pathways include the vagus nerve and the spinal cord, notably lamina I, which relay sensory signals from internal organs via specialized receptors. Additionally, structures including the CVOs and the enteric nervous system also contribute internal sensory information to the interoceptive system, integrating peripherally with afferent pathways or centrally within processing hubs [19,63]. The vagus nerve and spinal cord also contain efferent control branches to maintain internal homoeostasis and respond to physiological disturbances. Together, they sense internal conditions, maintain homoeostasis and construct dynamic, continuous maps of the organism [30]. While the interoceptive system can be divided into distinct branches, structures and pathways, it remains deeply interconnected, engaging in extensive peripheral and central communication and ultimately gives rise to the *feeling mind*.

The vagus nerve (cranial nerve X), from the Latin ‘*wandering nerve*’, innervates virtually all of the ‘milieu intérieur’, a term coined by French physiologist Claude Bernard to refer to our physiological interior. The right and left branches of the vagus nerve originate in the brainstem from the nucleus tractus solitarius (NTS) of the medulla oblongata, and project throughout the body, making abundant connections with vital internal organs, such as the heart, lungs, oesophagus, stomach, intestines, liver, gallbladder, immune tissues and parts of the colon [64,65]. As the main nerve of the parasympathetic nervous system, it regulates involuntary functions, including cardiac, respiratory, digestive and immune processes.

Still, the vagus nerve is predominantly sensory; 80% of its fibres are afferent and 20% efferent [66], allowing it to both *sense* and *regulate* homoeostasis. The afferent sensory neurons continuously sense internal states using specialized transduction structures, such as mechanoreceptors and chemosensors [26] (table 1), and then project centrally to the NTS. In mammals, around 80% of vagal fibres are unmyelinated, with the remainder being poorly myelinated [25,85]. As noted earlier, the axons are organized into Remak bundles, which results in the extreme close proximity of the constitutive fibres. All of these features facilitate NSS and allow a continuous and seamless neural–non-neural interface [86].

**Table 1.** Main sensory receptors in vagal afferents: function and anatomical location. The afferent neurons of the interoceptive system transduce widely different types of information to build dynamic maps of the internal milieu.

sensory receptors	function	anatomical locations
mechanoreceptors	detect visceral organ distension	gut [67], cardiovascular system [68], lungs [69] etc.
chemoreceptors	monitor chemical signals (e.g. pCO₂, pH, oxygen, insulin and glucose) to regulate chemical homeostasis	muscle [70], liver [71], adipose tissue [72], kidneys [73], brain [74], cardiovascular system [75], gut [76] etc.
metaboreceptors	sense metabolic byproducts (e.g. lactate and ATP) to signal muscle fatigue and energy demand	skeletal muscles [77], connective tissues [78] etc.
thermoreceptors	measure internal temperature	deep somatic tissue [79], gut [80] etc.
nociceptors	detect visceral pain, irritation and inflammation	oesophagus [81], lungs [81], cardiovascular system [82] etc.
osmoreceptors	monitor osmolality	gut [83], liver [84] etc.

Vagal afferents are pseudo-unipolar neurons with a peripheral branch that detects sensory stimuli and a central branch projecting to the NTS. Their cell bodies are located in the nodose ganglion or the jugular ganglion, where the peripheral axons from internal organs converge before continuing centrally to the brainstem.

The interoceptive system consists of distinct pathways with extensive crosstalk at various levels. For instance, the vagus nerve receives direct input from specific CVOs, such as the area postrema [59], yet does not depend on peripheral connections with the spinal cord [87]. Instead, spinal-receiving regions in the brainstem and cortex are highly interconnected with vagal central target regions [30]. Why would nature have indulged in this segregation? The likely answer lies in structural and functional specialization. The spinal cord primarily transmits signals related to pain, temperature and tissue damage—often via the sympathetic nervous system—while the vagus nerve carries ‘physiological-range signals’ from internal organs, primarily linked to parasympathetic functions [26]. *In brief, it appears that the vagus nerve fundamentally transmits physiological-range signals—and is thus involved in physiological regulation—whereas the spinal cord pathways are associated with time-sensitive alarm signals related to pathological states*. Further research is needed to clarify the functional distinctions between the various pathways of the interoceptive system [26,58].

As vagal axons ascend towards the brainstem, they maintain a ‘salt-and-pepper’ organization, preserving the structure of interoceptive signals while allowing extensive cross-modal NSS [88]. These inputs converge in the NTS of the medulla, where they are systematically mapped to form a detailed representation of the viscera [89] and are subsequently re-represented in visceral maps of the insular cortex [30]. Thus, the NTS serves as the first central hub for self-representation, highlighting the brainstem’s crucial role in contributing to the ‘self’ process and consciousness. This is a challenge for prevailing views that attribute consciousness to the activity of higher-order cortical regions, notably the insula, or to the general complexity of cortical interactions [90,91]. Instead, the brainstem-centred perspective we favour posits *consciousness as part of a neurobiological process dedicated to homoeostatic regulation, rooted in interoception and in lower-order brainstem structures*.

Although the vagus nerve is difficult to access, its vital role in homoeostatic feelings has made it a focal point of state-of-the-art research and clinical interest. Non-invasive therapeutic strategies, including vagus nerve stimulation, have confirmed its role in cardiovascular, respiratory, immune and digestive functions and internal pain [66,92–94]. These interventions have shown promise in treating neuropsychiatric disorders, including depression and anxiety. Of note, top-down regulation of homeostasis can also influence mood, feelings and, consequently, states of consciousness [95].

The vagus nerve is a key contributor to interoception, with unique anatomy and connectivity allowing real-time, graded and continuous communication with the body. By converging onto the NTS, it constructs dynamic maps of the internal milieu, supporting a continuous representation of the organism as a primary substrate for consciousness. While the vagus nerve is not the sole interoceptive afferent pathway, it does play a fundamental role in mapping internal states. Understanding how its afferent neurons collect, transmit and integrate information is important to uncovering how homoeostatic feelings support the process of consciousness.

## 5. The adaptive advantage of consciousness

### (a) Interoceptive feelings support the continuity of conscious experience

Our conception of consciousness proposes that the continuous stream of spontaneously conscious interoceptive feelings capably reflect the body’s current state, forming the de facto basis for an uninterrupted sense of self. Interoceptive feelings underpin the sense of direct ownership of the organism’s body.

By contrast, exteroception describes the world external to the body, including the voluntary movements we execute in that external world, thus providing information about our surroundings rather than about the self. With the exception of looking at yourself in the mirror or looking at your own body, the properties it registers are not constituents of the self. Exteroceptive sensations are episodic and event-driven, characterized by discrete, targeted, rapid and sharply defined signalling, typical features of digital processing, which enable efficient scanning of an uncertain and largely unknown environment.

Similarly, cognitive and linguistic processes describe the world through computation and simulation, generating high-level inferences, images and narratives from lower-level information. While invaluable for abstract reasoning, planning and communication, they are not directly essential to the moment-by-moment maintenance of the self’s integrity. Unlike interoception, they do not anchor bodily unity; instead, they build on it.

We therefore suggest that the *feeling mind* is the foundation upon which exteroception, cognition and language can operate and ultimately provide consciousness proper. Spontaneously conscious interoceptive, homoeostatic feelings are the basis for subjectivity. Consciousness proper results from the combination of subjectivity with the exteroceptive, cognitive and linguistic processes that constitute the *modern mind*.

Multimodal tasks are needed to probe the various dimensions of interoception [96]. A deeper understanding of the interoceptive system’s internal structure could help clarify how specific components give rise to distinct affective states. While some work has begun in this area [97–99], further research is essential.

### (b) Consciousness enables comprehensive homoeostatic regulation

*The subjectivity component of consciousness serves a clear evolutionary purpose: to generate a spontaneous sense of self and belonging*. This motivates the correction of homoeostatic deviations that threaten the continuation of life (e.g. hunger, pain), along with taking advantage of deviations that promise the maintenance and expansion of life (e.g. wellbeing). The comingling of the body proper with the nervous system transforms homoeostatic disturbances—such as hypoglycaemia, tissue damage or shifts in osmolality—into continuous and consciously felt experiences: hunger, pain and thirst, respectively. Such experiences convey critical information about the organism’s internal state, prompting complex neural responses that engage the external environment and restore balance. For instance, the subjective feeling of illness in response to infection arises spontaneously as a signal of disrupted internal homeostasis. Far from episodic, it is a persistent and continuous state of awareness, occupying the forefront of the conscious mind until the infection is resolved and homeostasis restored. Its intensity reflects the severity of the disturbance: the greater the infection, the stronger the felt imbalance—the feeling is graded. Feeling ill is a diffuse, multimodal experience, shaped by extensive crosstalk among interoceptive pathways. Energy wanes, body temperature rises, and widespread bodily discomfort compels the individual—now consciously aware of homoeostatic disarray—to adopt corrective and protective responses.

Because the brainstem’s interoception-dedicated nuclei are highly conserved across species [100–102], they provide a valuable phylogenetic opportunity for studying consciousness. In primates, the integration of brainstem interoceptive regions with higher-order cognitive functions enables forms of consciousness shaped not only by feeling but also by narrative, reasoning and memory—revealing a continuum of conscious experience.

## 6. Contrast with other theories of consciousness

In contrast to theories such as global neuronal workspace (GNW) [103,104], integrated information theory (IIT) [105] and higher-order thought (HOT) [106], we propose that interoceptive, homoeostatic feelings are the foundational substrate of consciousness. We recognize that such frameworks offer valuable insight into the richness and structure of complex conscious states—particularly in humans—as enhanced informational and cognitive capacities enrich experience. However, we believe that even highly integrated systems require a unifying representational anchor—homoeostatic feelings and a feeling self—for consciousness to emerge.

## 7. Artificial intelligence and natural intelligence

Currently, artificial intelligence (AI) excels at replicating the architecture and processing of exteroceptive, cognitive and linguistic systems, but it struggles to capture the dynamics of interoceptive processing. Most neural networks rely on discrete, rapid and precise stimulus–response learning, whereas the *feeling mind* operates at various timescales by integrating slow, continuous and graded information [107]. While digital signalling can sometimes produce analogue-like behaviours through complex—and often costly—recursive computations, it falls short of capturing the brain’s fundamentally analogue character. Recent work across theoretical neuroscience, software and electrical engineering is beginning to reverse this trend by incorporating analogue functions. A compelling example is the rise of ‘neuromorphic devices’—hardware designed to emulate biological processes. Among these, ‘memristive’ systems stand out for their ability to retain memory of prior signals, influencing future computations in a manner that mimics neuronal learning and echoes the function of subthreshold potentials in the brain [7]. The extensive, analogue-like interneuronal communication underlying interoception across multiple timescales requires more advanced models to capture nonlinearities and multidimensional processing. This effort grows increasingly costly with traditional digital systems.

Many AI architectures have difficulty incorporating the body’s continuous self-monitoring, where redundancy, adaptability and slow integration govern physiological regulation. Contemporary AI is not well suited to modelling the interoceptive mechanisms that anchor homoeostatic feelings and consciousness.

## 8. Conclusion

The interoceptive nervous system operates using fundamentally distinct processing mechanisms as compared with the exteroceptive, cognitive and linguistic systems. The former relies principally on analogue-like processes—which give rise to continuous and adaptive real-time maps of the internal milieu—whereas the latter rely predominantly on rapid, precise and point-to-point time-sensitive signalling.

We argue that these signalling differences arise from distinct neurobiological and anatomical features. The low myelination of afferent interoceptive neurons enables extensive non-synaptic signalling throughout interoceptive pathways, notably in the tightly bundled axons of the vagus nerve. Key interoceptive organs devoid of a blood–brain barrier allow direct communication between the interoceptive nervous system and the body proper. These features establish a seamless interface between brain and body—in effect a blended, comingled interface—giving rise to dynamic and continuous real-time maps of the internal milieu, which are at the core of homoeostatic regulation and constitute subjectivity, the foundation of consciousness. Consciousness serves an adaptive purpose by allowing homoeostatic deviations to be spontaneously felt and by enabling the organism to coordinate complex interactions with its environment—guided by exteroception and higher-order processes—to restore or exploit homoeostasis.

The intricacies of interoceptive signalling are largely absent from current artificial intelligence designs, identifying a fundamental gap between the processes of artificial and natural intelligence. Analogue signalling is often dismissed in computational neuroscience, a blind spot that frequently leads to inefficient brain-inspired algorithms, limited neuromorphic design and a narrow computational account of higher-order cognition [108]. While substantial progress is underway, the full computational complexity of the human brain remains irreproducible with the current technology.

## Data Availability

This article has no additional data.
